# Active virus-host interactions at sub-freezing temperatures in Arctic peat soil

**DOI:** 10.1186/s40168-021-01154-2

**Published:** 2021-10-18

**Authors:** Gareth Trubl, Jeffrey A. Kimbrel, Jose Liquet-Gonzalez, Erin E. Nuccio, Peter K. Weber, Jennifer Pett-Ridge, Janet K. Jansson, Mark P. Waldrop, Steven J. Blazewicz

**Affiliations:** 1 Physical and Life Sciences Directorate, Lawrence Livermore National Laboratory, Livermore, CA USA; 2 Department of Life and Environmental Sciences, University of California, Merced, CA 95343 USA; 3 Biological Sciences Division, Pacific Northwest National Laboratory, Richland, WA USA; 4 U.S. Geological Survey, Geology, Minerals, Energy, and Geophysics Science Center, Menlo Park, CA USA

**Keywords:** Soil viruses, Bacteriophage, Stable isotope probing, Permafrost, Peat, ^18^O-water, Metagenomics

## Abstract

**Background:**

Winter carbon loss in northern ecosystems is estimated to be greater than the average growing season carbon uptake and is primarily driven by microbial decomposers. Viruses modulate microbial carbon cycling via induced mortality and metabolic controls, but it is unknown whether viruses are active under winter conditions (anoxic and sub-freezing temperatures).

**Results:**

We used stable isotope probing (SIP) targeted metagenomics to reveal the genomic potential of active soil microbial populations under simulated winter conditions, with an emphasis on viruses and virus-host dynamics. Arctic peat soils from the Bonanza Creek Long-Term Ecological Research site in Alaska were incubated under sub-freezing anoxic conditions with H_2_^18^O or natural abundance water for 184 and 370 days. We sequenced 23 SIP-metagenomes and measured carbon dioxide (CO_2_) efflux throughout the experiment. We identified 46 bacterial populations (spanning 9 phyla) and 243 viral populations that actively took up ^18^O in soil and respired CO_2_ throughout the incubation. Active bacterial populations represented only a small portion of the detected microbial community and were capable of fermentation and organic matter degradation. In contrast, active viral populations represented a large portion of the detected viral community and one third were linked to active bacterial populations. We identified 86 auxiliary metabolic genes and other environmentally relevant genes. The majority of these genes were carried by active viral populations and had diverse functions such as carbon utilization and scavenging that could provide their host with a fitness advantage for utilizing much-needed carbon sources or acquiring essential nutrients.

**Conclusions:**

Overall, there was a stark difference in the identity and function of the active bacterial and viral community compared to the unlabeled community that would have been overlooked with a non-targeted standard metagenomic analysis. Our results illustrate that substantial active virus-host interactions occur in sub-freezing anoxic conditions and highlight viruses as a major community-structuring agent that likely modulates carbon loss in peat soils during winter, which may be pivotal for understanding the future fate of arctic soils' vast carbon stocks.

Video abstract

**Supplementary Information:**

The online version contains supplementary material available at 10.1186/s40168-021-01154-2.

## Introduction

Northern peatlands are important terrestrial ecosystems for carbon (C) storage, estimated to contain one-third of soil C (~1000 gigatons) [1], yet the fate of this C is unknown as these soils are experiencing dramatic changes from anthropogenic climate change [2–4]. While soil-warming experiments indicate increased carbon dioxide (CO_2_) and methane emissions under global climate change, it is likely that these C losses from northern peatlands are underestimated because virtually all measurements neglect winter processes [5–8]. Recent estimates show substantial winter C loss [9], which may be greater than the average growing season C uptake [10]. While the winter months include large air temperature fluctuations and extreme temperature minimums [11], the temperatures found in much of the soil profile of permafrost or seasonally frozen bogs can remain stable and just below the freezing point (−1 to −5°C) [5, 12, 13]. Bacteria have been shown to remain active below the freezing point in soils with both catabolic and anabolic activities observed [14–16]. Activity is likely facilitated by a portion of the water remaining liquid at temperatures below 0°C with evidence that more than 20% of the water can remain unfrozen in peat soils incubated between −1 and −5°C [17]. Therefore, it is critical to understand the identity, functional capacity, and activities of bacteria and viruses that cause C turnover in frozen soils to better predict their biogeochemical implications.

In soils, viruses may play a major role in microbial population dynamics, genome evolution, and the fate of soil C [18]. In marine systems where viruses are well-studied, viruses kill ~40% of bacteria daily and sustain up to 55% of new bacterial C production [19, 20]. Soils hold an enormous viral reservoir, but we know remarkably little about their diversity, activity, host interactions, and persistence as compared to other environments [18, 21]. Viruses can affect soil C cycling by stopping microbial metabolism via cell death during lytic infections and releasing cell-derived nutrients into the environment [22]. These nutrients can either fuel other organisms’ metabolism or be stabilized via entombing effects [23]. This process can be prolonged by temperate viruses, which can undergo lysogenic infection and remain latent in their host for long periods of time. During lysogenic infection, viruses still impact host metabolism via host gene regulation and acquisition of new virulence factors, but it is primarily for maintaining the provirus in the host genome [24]. Temperate viruses will eventually switch to either lytic infection or chronic infection, in which viruses are slowly shed from the cell over time without cell death. Viruses can also carry auxiliary metabolic genes (AMGs), which are host-derived genes that can be expressed during infection and typically aid the infection process by overcoming energetic limitations [25, 26]. What proportion of soil viruses carry AMGs and whether these AMGs are actively expressed in microbial hosts remains unknown [24]. These are especially arduous tasks because most viruses are detected in metagenomic datasets as partial genome fragments and can have microbial contamination (44). Inroads into AMG detection are being made using meta-omic approaches [27] combined with benchmarked bioinformatics [28, 29] and, in marine environments, tractable virus-host model systems. Recently, some of these techniques have been applied to characterized viruses in northern peatlands during the growing season. These studies indicate that viruses are largely unrelated to other known viruses, highly diverse, endemic to their habitat, infect dominant microbial populations, and carry AMGs that could impact C cycling [30, 31]. The question of whether viruses are active during the winter (i.e., the seven to nine months of the year when Arctic peatlands are frozen) remains unanswered.

Stable isotope probing (SIP) combined with metagenomics is an effective tool for tracking the active microbial taxa in complex communities, linking individuals to specific functions [32–34], or characterizing specific microbes and the viruses that infect them [35]. While many isotope tracing studies use ^13^C-enriched tracers (e.g., ^13^CO_2_, ^13^C-plant biomass, or ^13^C-glucose) to identify specific substrate degraders [33] or assess microbial C use efficiency [36], heavy-water (H_2_^18^O) SIP-metagenomics has the unique benefit of labeling all actively growing microbes because water is a universal substrate for nucleic acid synthesis [37–39]. Over adequate time scales, this technique is sensitive enough to label slow-growing or less abundant microbes and identify taxon-specific microbial growth and mortality patterns [40–43], but heavy-water SIP-metagenomics has not been used to label active viruses.

We hypothesized that viruses continue to infect and replicate during winter months even when the soils reach below freezing temperatures, and their activities affect microbial growth, mortality, and C cycling. By characterizing the identity of active viruses, who they infect, what their genetic repertoire is, and their temporal dynamics, we can begin to understand their importance in regulating microbial biogeochemistry and nutrient flow in soils during winter. We used heavy-water SIP-metagenomics to label actively replicating microbial taxa and viruses and determine whether viruses play a role in controlling active microbial population dynamics and metabolic function in peat soil under simulated winter (sub-freezing and anoxic) conditions. To our knowledge, viral activities have never been confirmed under such conditions, and understanding their impact on microbial activity and soil C transformation during winter months may be pivotal for understanding the future fate of the vast organic C stocks in Arctic peatlands.

## Methods

### Experimental setup

We collected soil samples from the Alaska Peatland Experiment (APEX) site, part of the Bonanza Creek Long Term Ecological Research site southwest of Fairbanks, Alaska (64.70 °N, −148.3 °W), in a zone of discontinuous permafrost (overview of methods in Fig. S1). Three peat soil cores were collected from the active layer of a *Sphagnum*-dominated thermokarst bog on June 16, 2013, from the APEX beta site approximately 20 m south of the flux tower. During the week of sampling, midday air temperatures were 27°C, midday surface (0–10-cm depth) peat temperature was 10°C, active layer depth in the bog was 35 cm, and the water table was 3–8 cm below the surface. The pH was 4.80 (1:1 soil to water ratio). Vegetation and other soil and flux data are given in Waldrop et al. [9]. To collect the cores, moss and peat were removed down to the top of the water table using scissors. The top 20 cm below the water table were collected using a 6.3-cm diameter sharpened steel barrel corer attached to an electric drill. Three cores were collected 1 m apart along a transect. The cores were stored in mason jars filled with porewater collected from the core hole using a 60-ml syringe fitted with Tygon tubing. Sealed jars were immediately shipped on ice to the U.S. Geological Survey (Menlo Park, CA, USA) where they were homogenized in an anaerobic glovebox maintained at 4°C with oxygen continuously maintained at 0 ppm using a N_2_ headspace containing H_2_ (5%) to react with a palladium catalyst and monitored by an anaerobic monitor (CAM-12, Coy Lab Products). All processing of the soil was completed in the anaerobic chamber until they were sealed in incubation vials. Soil subsamples (2 g of soil wet weight) were collected from each core, pressed to remove porewater (using a 5-ml syringe fitted with a nylon screen and a glass fiber filter), and transferred to Wheaton serum vials (10 ml), creating 12 incubation vessels. Anoxic synthetic porewater was created by freeze-drying filtered (0.45 μm polytetrafluoroethylene then 0.2-μm nylon filter) soil porewater collected in tandem with the cores, and then rehydrating the remaining particulates with either heavy water (97 atom% H_2_^18^O, Cambridge Isotope Laboratories) or natural abundance water (control). Molecular oxygen was removed from the synthetic porewater using standard anaerobic culturing methods [44, 45] by bringing the solution to a boil while stirring under a steady stream of N_2_ (5.0 ultra-high purity N_2_ filtered through a Restek high-capacity oxygen trap was used for all setup and gas collection steps outlined in the methods), then quickly cooling in ice water while under a continues flow of N_2_, and then bottles were sealed with blue butyl rubber septa. Anoxic synthetic ^18^O-enriched porewater was added (2.5 ml) to half of the incubation vials, and anoxic synthetic natural abundance porewater was added to the other half using a 5-ml gas-tight syringe with a 23-G needle that was purged with N_2_. All the sample vials were submerged in a glycerol bath at 4°C, and the temperature was slowly and steadily reduced to −1.5°C, over 48 h. Samples were then continuously maintained at −1.5°C for 184 days (midyear) and 370 days (full year). At each timepoint, biological replicates (*n*=3) were destructively harvested and snap frozen in liquid N_2_ and stored at −80°C. Six parallel samples were set up in a similar manner for headspace gas analysis to quantify CO_2_ production, except these used proportionally larger amounts of soil (18.15 g wet soil) and larger incubation vials (100 ml), and three of the gas production vials were incubated at −20°C as a control.

### CO_2_ production quantification

Gas samples were collected from the gas production vials at 10 timepoints over the 370-day incubation. To prevent oxygen from contaminating the incubation vials, a 5-ml syringe with a stopcock and 23 G needle was cleared 3 times with O_2_-free N_2_. The syringe was then inserted into the vial septa and plunged 3 times to mix the headspace; 2 ml headspace was collected, and the stopcock locked. The 2 ml samples were transferred to 10 ml serum bottles that had been purged and then filled with N_2_ (1 atm). Gas samples were analyzed via gas chromatography equipped with a methanizer paired with a flame ionization detector (SRI 8610GC, SRI Instruments, Torrance, CA) to quantify headspace CO_2_.

### DNA extraction and density gradient SIP

DNA was extracted from each replicate using a modified phenol/chloroform protocol [46]. In summary, 0.5 g (+/− 0.01 g) wet soil was added to lysing matrix E tubes (MilliporeSigma, Burlington, MA), and 100 μl 1x TE (pH 7.5), 150 μl PO_4_ buffer (0.2 M in 1 M NaCl), and 100 μl 10% SDS were added and vortexed. Tubes were bead beat for 30 s at 30 1/s and briefly centrifuged. 0.6 ml phenol:chloroform:isoamyl alcohol (25:24:1) was added, vortexed, and incubated at 65°C for 10 min. Tubes were spun for 5 min at 10K × g, and the supernatant was transferred to a new tube. The bead beat tubes were then re-extracted using 220 μl 1x TE and 80 μl PO_4_ buffers. The supernatant from the first and second extracts was combined in a new 2 ml tube. 550 μl phenol/chloroform/isoamyl alcohol was added, mixed, and centrifuged (10K × g, 5 min). The aqueous top layer was transferred to a new 2 ml tube. 900 μl chloroform:isoamyl alcohol (24:1) was added, mixed, and centrifuged (10K x g, 5 min), and the supernatant transferred to a new 2 ml tube. Then, 850 μl chloroform:isoamyl alcohol (24:1) was added, mixed, and centrifuged (10K x g, 5 min), and the supernatant transferred to a new 1.7 ml tube. RNAase was added (6.44 μl, 10 mg/ml), mixed, and incubated at 50°C for 10 min. 244 μl 10 M ammonium acetate was added, mixed, and incubated at 4°C for 2 h. Tubes were centrifuged at 16.1K x g for 15 min, and the supernatant transferred to a new tube. Isopropanol (670 μl) was added, mixed, and centrifuged (16.1K x g, 20 min). The supernatant was removed, and the DNA pellet dried in a PCR hood for 15 min. 30 μl 1x TE was added and mixed, and the DNA then stored at −80°C.

Extracted DNA was fractionated via CsCl density gradient ultracentrifugation to separate ^18^O-enriched DNA as described previously [40]. DNA was binned into 5 fractions based on density, and the binned DNA from the two heaviest fractions (medium-heavy [MH; 1.717–1.725 g/ml] and heavy [H; 1.725–1.750 g/ml]) was sequenced separately.

### Sequencing and metagenome generation

DNA from the SIP fractions was sent to the Keck Sequencing Facility at Yale University. For each sample, and 100 ng of DNA was sheared to 500 bp using the Covaris E210 (Covaris, Inc., Woburn, MA, USA), followed by a SPRI bead cleanup using Ampure XP (Beckman Coulter Life Sciences, Brea, CA, USA); the DNA quality was checked using a Bioanalyzer chip. The sheared gDNA from the 24 samples (3 reps x 2 isotopes x 2 timepoints x 2 density fractions) was then end-repaired, A-tailed, adapters ligated, and sequenced on an Illumina HiSeq 2500 to generate metagenomes (Table S1). Paired-end 151 nt reads were processed in three steps with bbduk v38 (Bushnell, B.): (1) adapter-trimming (ftl=10 ktrim=*r k*=23 mink=11 hdist=1 tpe tbo minlen=50), (2) PhiX and Illumina adapter/barcode removal (*k*=31 hdist=1 minlen=50), and (3) quality-trimming (qtrim=*r* trimq=10 minlen=50). Metagenomes were successfully generated for 23 of the samples (one did not have enough DNA recovered), with 302 Gbp of sequencing data (Table S1).

### Recovery of vOTUs from metagenomes

Virus-specific informatics were used to increase the number of viral sequences detected in these soil datasets [18]. Processed reads were assembled into contigs using SPAdes v3.11.1 (--only-assembler --phred-offset 33 --meta -k 25, 55, 95 )[47]. From the 23 SIP-fractionated metagenomes, we assembled 51,487,619 contigs, with 63% of the total reads mapped to the contigs. Contigs were processed with VirSorter (virome decontamination mode) [48] and DeepVirFinder (DVF) [49] to detect dsDNA viral contigs. ssDNA and RNA viruses were not investigated in this study but should be considered in future studies. We retained contigs that were ≥10 kb, sorted into VirSorter categories 1 and 2, and had a DVF score ≥0.9 and *p* value <0.05. Viral contigs were clustered at 95% average nucleotide identity (ANI) across 85% of the shorter contig [50] using nucmer [51] to generate a nonredundant set of viral populations (vOTUs; Table S2). vOTU quality was assessed with CheckV (default parameters) [52]. Coverage of the vOTUs was estimated based on post-quality-controlled read mapping at ≥90% ANI and covering ≥75% of the contig [50] using Bowtie2 [53]. Coverage was then normalized per gigabase-pair of metagenome and by the length of the contig [54]. The activity of vOTUs was determined by a vOTU being present in the H_2_^18^O samples and not present in the paired natural abundance water samples. Diversity metrics for the active vOTUs were calculated using the Vegan package in R, and a *t* test was used to determine a significant difference between the midyear and full-year time points. Outliers were first identified by finding the difference between the first and third quartile of the distribution and multiplying it by 2.2 [55, 56]. The vOTUs were annotated using the virus-centric multiPhATE pipeline (default parameters) [57] and the AMG-centric DRAM-v pipeline (with --skip_uniref) [28]. We note that DRAM-v provides AMG scores only for vOTUs detected via VirSorter; AMGs predicted from the 208 vOTUs recovered from DVF were manually curated. For this manual inspection, we removed putative AMGs that were at contig ends or those with annotations from multiple functional categories. To determine the proportion of temperate vOTUs, we searched for genes associated with proviruses, such as integrase and parA [24], leveraged classification from VirSorter (categories 4 or 5) and our genome-similarity host linkages (≤90% similarity), and used two tools—CheckV [52] and PHASTER [58]. Temperate viruses were labeled as proviruses if they matched a linked MAG (≤90% of a contig in a microbial genome bin) or were classified as one by VirSorter, CheckV, or PHASTER. A list of metadata for vOTUs [50] is provided in Table S3.

### MAG curation and host linking

To generate metagenome-assembled genomes (MAGs), read-pairs from the biological replicates were grouped for 8 separate co-assemblies (2 timepoints x 2 SIP fractions x 2 O isotopes) with MEGAHIT v1.1.4 (--k-min 27 --k-max 127 --k-step 10) [59]. Contigs ≥1 KB were separately binned with Concoct v1.0.0 [60], MaxBin v2.2.6 [61], and MetaBAT v2.12.1 [62]. Genome bins from these three binning tools were refined using the bin_refinement module of MetaWRAP v1.2.1 (-c 50 -x 10) [63] with CheckM v1.0.12 [64], using the CPR marker set [65]. Only genome bins with at least “medium quality” according to MIMAG standards [66] were retained.

Two methods were used to define MAGs as active: (1) a read-subtraction approach and (2) a log-fold-change approach. For the read-subtraction approach, contigs from the ^16^O assemblies were used as a reference to subtract the ^18^O reads that aligned with the unlabeled dataset using bbsplit v38 (maxindel=1) [67]. The isotopically labeled reads that did not align with the unlabeled dataset were considered “active” reads and were then processed through the same MAG assembly workflow described above (starting with the MEGAHIT assembly through MetaWRAP refinement); this generated genome bins reconstructed from distinct ^18^O reads from the two SIP fractions at two timepoints. Refined bins from all 12 groups (8 total + 4 active) were dereplicated into a final set of MAGs using dRep v2.2.3 (-comp 50 -con 10 -p 6 -nc 0.6) [68]. MAG taxonomy was determined using GTDB-tk v0.3.2 [69] with the version r89 Genome Taxonomy Database (GTDB; https://gtdb.ecogenomic.org/). Structural annotation was done using Patric [70], and general functional annotation with RASTtk [71] for subsystems within Patric v3 (retaining subsystem variants predicted to be active or likely), and KofamScan v1.1.0 [72] with the KEGG [73] 93 database. Per-sample MAG abundances were determined by aligning each sample’s filtered reads against the MAG genomes using bbmap v38 [67] to obtain total assigned reads per contig and average fold coverage per contig.

For the log-fold-change approach to define active MAGs, we assessed significant (5% false-discovery rate) and positive log2-fold change in ^18^O versus ^16^O read abundances within a time point and SIP fraction. Fold changes were determined using wrench-normalized [74] total assigned reads per MAG with a zero-inflated log-normal model implemented in metagenomeSeq [75].

The vOTUs and MAGs were linked via clustered regularly interspaced short palindromic repeats (CRISPR) spacers and shared genomic content as previously described [76]. Briefly, CRISPR regions were detected in the MAGs using MinCED (options -minNR 2 -spacers; allowing 1 mismatch) [77] and linked to the vOTUs with BLASTn (options -max_target_seqs 10000000 -dust no -word_size 7) [78]. In addition, BLASTn (options -dust no -perc_identity 70) was used to link vOTUs and MAGs based on shared genomic content [79]. Virus-host abundance estimates were made by summing microbial host abundances at the phylum level in each timepoint and linked vOTU abundances. For linear regression, we assumed the virus abundance relied on the host. To confirm this, we only used abundances of viruses that we linked to hosts (using only these host abundances as well). Outliers were determined using the same permissive calculation as used for the *t* tests, and the goodness of fit for the linear regression was determined using the *R*^2^ coefficient and the coefficient *p* values for significance.

## Results

### Characterization of viruses

**Fig. 1:**
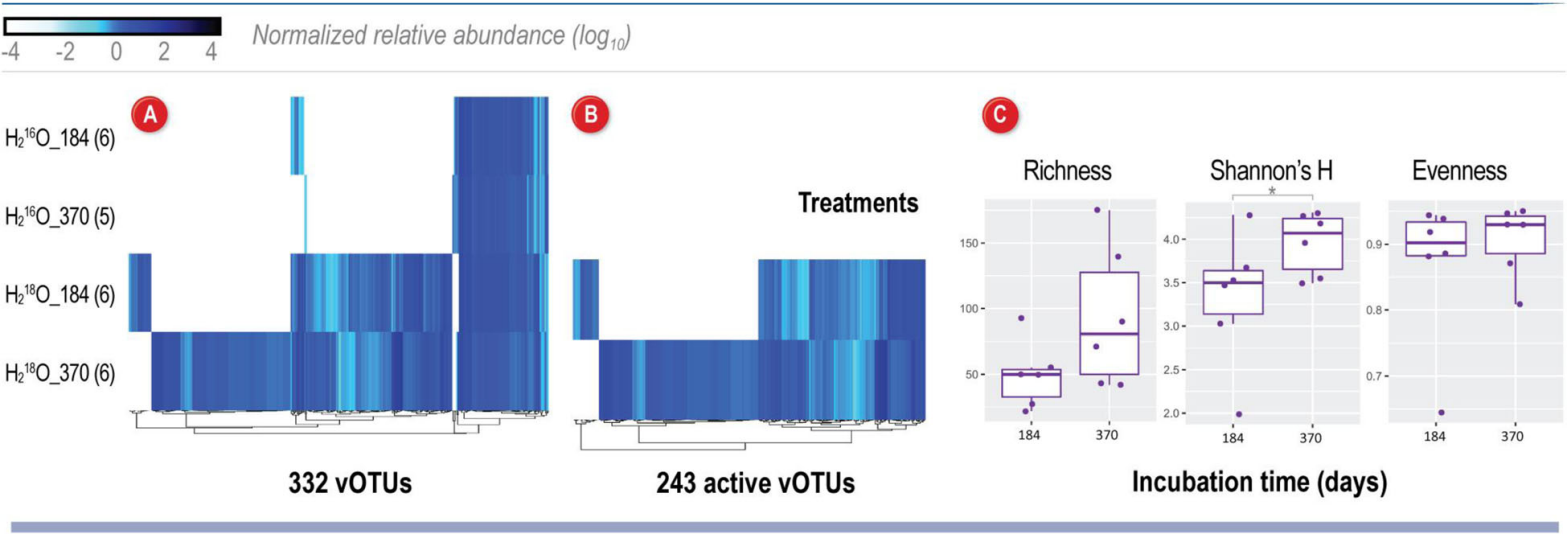
Assessment of viral community structure and activity after a ^18^O-water incubation in Arctic peat soils. **A** Viral sequences were identified from 23 samples grouped by two treatments (natural abundance water “H_2_^16^O,” and heavy water “H_2_^18^O”) and two time points: 184 days and 370 days. The number of replicates is indicated in parentheses. Relative abundances of all 332 vOTUs identified in the peat soils, clustered by abundances in each treatment/timepoint. **B** Relative abundances of 243 vOTUs considered “active” due to DNA ^18^O enrichment patterns. Relative abundance for each vOTU (illustrated by blue gradient) was normalized by metagenome size (total base pairs) and contig length, and reads were mapped to the contig if they shared ≥90% average nucleotide identity and covered ≥75% of the contig. **C** Diversity metrics for the 243 active vOTUs. Box plots show the median, upper, and lower quartile range, and the variance among the samples. Difference between midyear and full-year samples was determined with a *t* test and significance denoted by an asterix (*p* <0.05)

**Fig. 2:**
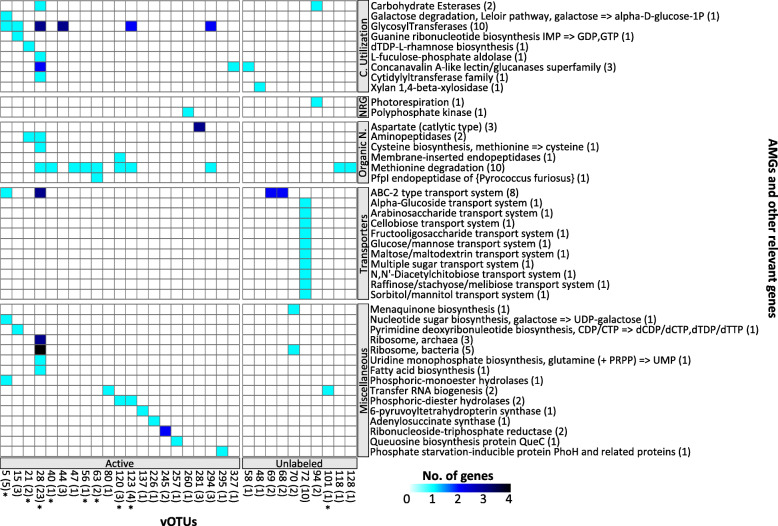
Auxiliary metabolic genes (AMGs) and other environmentally relevant viral genes. Heatmap of 31 vOTUs carrying confidently predicted AMGs and other environmentally relevant genes (Dram-v score 1–3) and their annotation. The vOTUs are grouped by active or unlabeled with the sum of the genes per vOTU indicated in parenthesis and those with an asterisk are linked to an active MAG. Genes are grouped by functional category—carbon utilization, energy generation, organic nitrogen use, transporters, and miscellaneous (see Table S6 for additional detail)

To characterize viral activity in Arctic peat soil under winter conditions, we analyzed viral sequences from heavy-water SIP-targeted metagenomic reads. Using two viral detection methods, we identified 5737 viral fragments (≥5 kb) that clustered into a nonredundant set of 332 vOTUs ≥10 kb (Table S3) that span 66 viral genera (see supplementary text; Fig. S2; Table S4). The size range of these vOTUs was 10,039–437,858 bp (average 32,954 bp) with 15 vOTUs ≥100 kb, including 5 so-called “jumbo” viruses (i.e., ≥200 kb) [80]. The vOTUs were well-covered (Fig. 1A) with an average of 17x coverage per metagenome, but with a large range, 3–147x. After quality checks, we identified 58 medium–high-quality vOTUs, of which four were considered “complete” according to community standards [50]. Genome quality could not be assessed for 93 vOTUs because they did not possess a known viral gene and were detected via a reference-free machine learning method [49]. Annotation of the vOTU genomes with the virus-centric multiPhATE pipeline predicted 15,772 genes, of which 61% were novel (Table S5). With the AMG-centric pipeline DRAM-v, we predicted 86 putative AMGs (score 1–3) and other environmentally relevant genes distilled into five functional categories (C utilization, energy, organic nitrogen, transporters, and miscellaneous) from 31 vOTUs (Table S6); 21 of the vOTUs were active and carried 63 AMGs and other environmentally relevant genes (Fig. 2). To identify temperate viruses, we searched the annotations for genes used in the lysogenic infection cycle and predicted nearly half (43%) of our vOTUs were temperate viruses. More than half (59%) of these temperate viruses were active, and the majority (80%) had at least one member of their population integrated at the time of sampling (provirus; Table S7).

Significantly more vOTUs were observed in the SIP fractions from heavy-water incubations relative to control (natural abundance water) incubations, confirming that many viruses incorporated ^18^O into their DNA (*p* < 0.05; Fig. S3). A majority (73%) of the vOTUs found in the high-density ^18^O SIP fractions were active at some point over the 370-day incubation, with about half active the full year and the other half only active in 184-day or 370-day samples (Fig. 1B). We measured active vOTU diversity to assess changes in the viral community structure from midyear to a full year of incubation. The Shannon’s H metric indicates a significantly (*p* ≤0.05) more diverse viral community at 370 days compared to 184 days (Fig. 1C). Shannon’s H diversity, which includes both richness and evenness, was driven by an average increase of more than 60% for vOTU richness from 184 to 370 days. Of these, 64 vOTUs became more abundant from 184 to 370 days, 110 became newly active, and 18 were no longer detected as labeled at 370 days.

### Host characterization based on SIP metagenomics

**Fig. 3:**
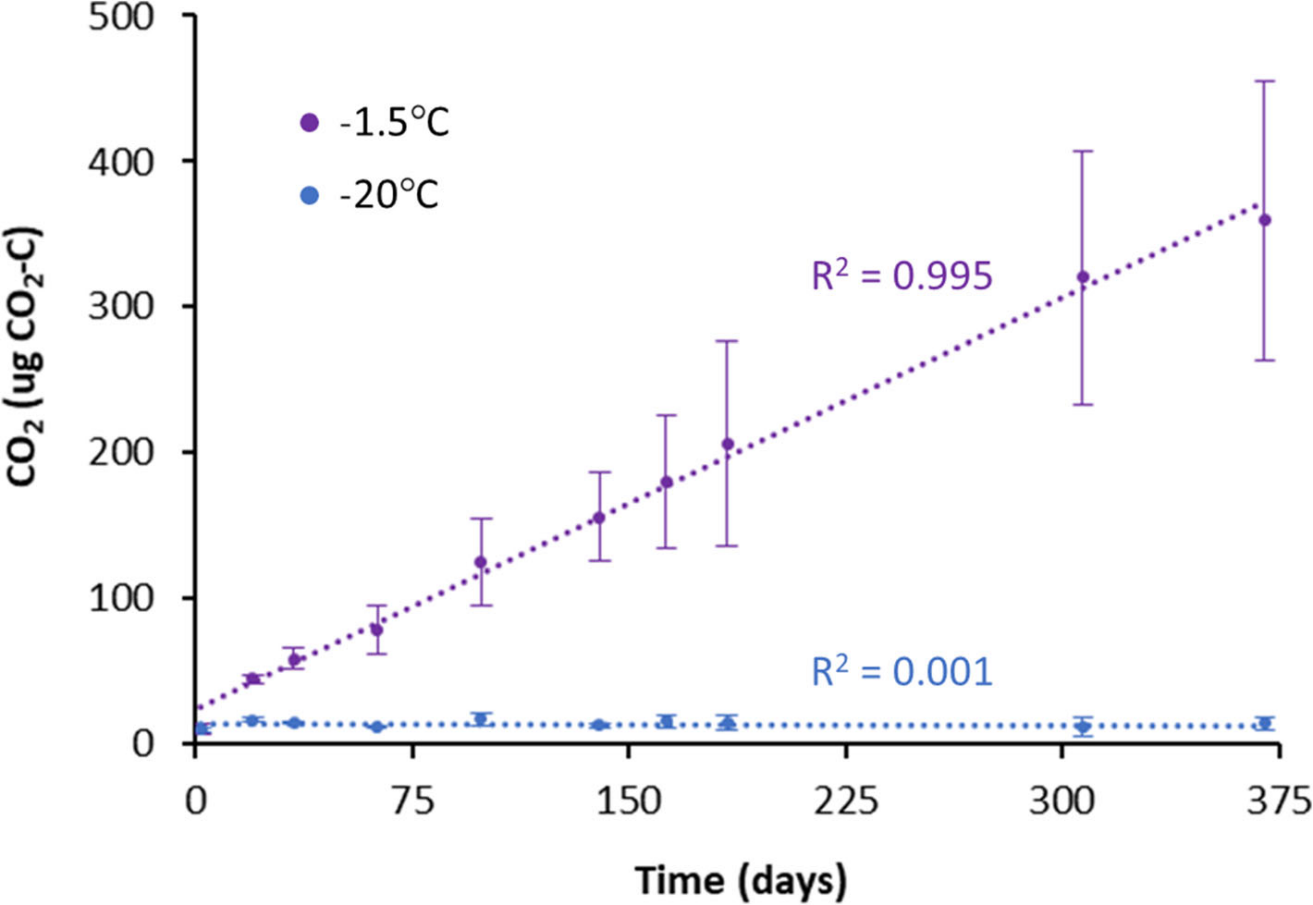
CO_2_ production. Cumulative CO_2_ production in soil incubated at −1.5°C (experimental conditions) and −20°C (control). Error bars show standard error (*n*=3), and *R*^2^ is shown for each linear regression

To identify potential viral hosts, we used a suite of metagenome assembly and binning methods which yielded 153 medium to high-quality MAGs, spanning 16 bacterial phyla, and 1 archaeal phylum (Table S8; GTDB taxonomy). The dominant phyla detected were Chloroflexota, Acidobacteriota, and Myxococcota (formally of the Proteobacteria). Incubation with heavy water indicated 30% (46) of these MAGs were actively growing, spanning 9 bacterial phyla, with the most abundant active populations from Acidobacteriota, Bacteroidota, and Firmicutes. By sequencing both unlabeled and isotopically labeled samples, we gained insight into genetic repertoire from both active and inactive bacteria (Table S9) but focused our efforts on characterizing the active bacterial lineages and metabolisms that viruses may be altering over winter months. We used CO_2_ production measurements to confirm microbial activity and C mineralization occurred continuously throughout the −1.5°C incubations, but not from the −20°C control incubations (Fig. 3).

### Linking vOTUs to MAG hosts

**Fig. 4:**
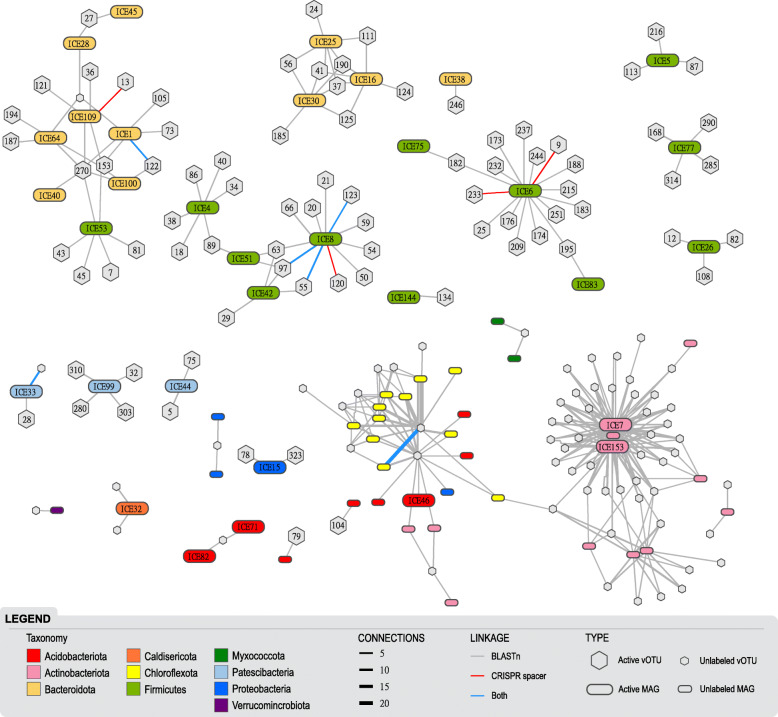
Virus-host linkages in Arctic peat soils incubated with ^18^O-enriched water. Network diagram illustrating vOTUs and their predicted bacterial hosts, colored by bacterial phyla. Active versus inactive vOTUs and MAGs (based on ^18^O enrichment) are indicated by large named or small hexagon/rounded rectangles, respectively. Lines represent linkages between a vOTU and a bacterial MAG, thickness denotes the number of connections, and are colored by the identification approach used: similarity in genomic content (gray), CRISPR spacer match (red), or both (blue). Additional details in Table S10 and Table S11

To understand the influence of the viruses on microbial dynamics during the winter season, we predicted potential microbial hosts via two different in silico approaches based on sequence similarity. First, we identified 11,879 CRISPR spacers in 136 of the MAGs and used these to link 10 vOTUs to 6 MAGs from 4 bacterial phyla (Fig. 4, Table S10). Leveraging the SIP activity patterns, we found most of these identified linkages connected active vOTUs and active MAGs (12 linkages between 8 active vOTUs and 5 active MAGs, 1 linkage between an unlabeled vOTU and an active MAG, and 3 linkages between unlabeled vOTUs and unlabeled MAGs). In a second approach, we used vOTU-MAG genome sequence similarity and recovered 798 similarity matches that linked 141 vOTUs to 65 MAGs from 10 bacterial phyla (Fig. 4; Table S11). Combined, the two virus-host linkage approaches indicated 318 unique connections between 145 vOTUs and 65 MAGs spanning 10 bacterial phyla. A significantly higher proportion of these vOTU-host matches were between active populations (Table 1). Notably, four vOTUs (#s 153, 161, 162, and 270) had a broad-host range and were linked to bacteria from several bacterial phyla (three of these vOTUs infected two phyla and one infecting four phyla; Fig. 4). Two of these broad-host-range vOTUs (153 and 270) were active and linked to only active MAGs from bacterial phyla Bacteriodota and Firmicutes.

**Table 1:** Linkages between ^18^O-labeled and unlabeled viruses and bacteria in SIP incubations

Group	Description	No. of vOTU-MAG matches	Unique vOTU-MAG matches	vOTUs	MAGs
1	Active vOTU-active MAG	164	107	74	37
2	Active vOTU-unlabeled MAG	2	2	2	2
3	Unlabeled vOTU-active MAG	226	77	44	11
4	Unlabeled vOTU-unlabeled MAG	422	132	58	30
	Total	814	318	178	80

Viral operational taxonomic units (vOTUs) were linked to metagenome assembled genomes (MAGs) via nucleotide similarity and CRISPR spacers. Active vOTUs and MAGs were defined based on assimilation of ^18^O enriched water (heavy water) into DNA. In total, there were 814 linkages from active vOTUs to active MAGs (group 1), active vOTUs to unlabeled MAGs (group 2), unlabeled vOTUs to active MAGs (group 3), and unlabeled vOTUs to unlabeled MAGs (group 4)

**Fig. 5:**
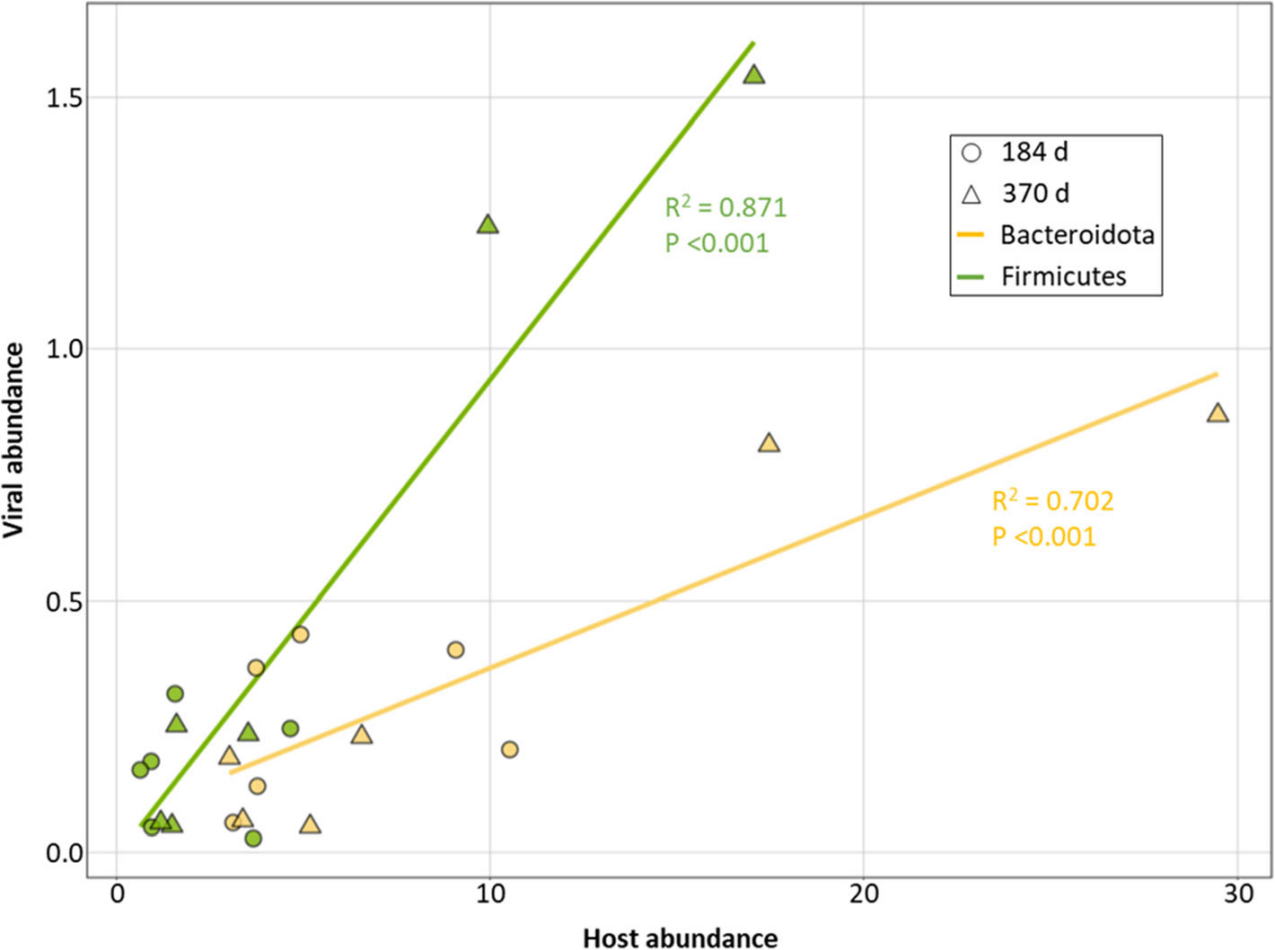
Abundances of active viruses and their predicted active bacterial hosts. Average virus:host abundance ratios for bacterial phyla Bacteroidota (yellow) and Firmicutes (green) from heavy-water treatment samples at 184 (*n*=6) and 370 days (*n*=6). Host abundance and the abundance of viruses for that host were calculated as the mean coverage depth from metagenomic read mapping, normalized by the number of reads in the sample. The goodness of fit for each linear regression line was determined using the *R*^2^ coefficient and associated *p* values (*p* <0.05). Additional information for viral abundances in Table S3 and for host abundances in Table S8

All 145 of the vOTUs we identified as host-linked may therefore be classified as dsDNA bacteriophages (since the vOTUs were linked to MAGs with a bacterium taxonomic assignment). These represented the majority (88%) of the host-virus matches, and almost all (92%) of the unlabeled vOTUs that were linked to unlabeled MAGs. In our soil incubations, Actinobacteriota (56%), Chloroflexota (24%), and Firmicutes (12%) were the most “infected” bacterial phyla (i.e., with the most vOTU-MAG linkages). Firmicutes (55%), Bacteroidota (34%), Patescibacteria (9%), and Proteobacteria (2%) were the only phyla that had active MAGs linked to active vOTUs. Of the active populations, 81 (33%) vOTUs and 33 (51%) MAGs were linked, with the top 15% most abundant active vOTUs predicted to infect Firmicutes and Bacteroidota, and the abundances of both these vOTUs and their host populations increased over the year incubation (Fig. 5).

## Discussion

Quantifying and predicting the drivers of C loss in northern latitude peatlands underlain with permafrost is a complex and critical issue, especially as these environments continue to be disproportionately impacted by climate change. Microbes largely govern C release from Arctic soils [4, 6, 10, 81, 82], and recent work has highlighted the important role viruses could play in microbial C processing during the growing season [30, 31]. However, in Arctic soils, very little is known about which microbes are active and virtually nothing is known about which viruses are active, how the viral community dynamics change, and how these viruses may modulate microbial biogeochemistry during winter. We address this gap in knowledge, using heavy-water SIP-metagenomics to directly identify active microbes and viruses and characterize their dynamics over a year under simulated winter conditions (−1.5°C and anoxic).

### Viral activity in cryoecosystems

Over the course of a year, we identified hundreds of active vOTUs, in stark contrast to the general paradigm that sub-freezing soils have little to no activity [83]. A third of these active vOTUs were linked to MAGs from active populations, highlighting that not only are microbes active in these anoxic sub-freezing temperature (−1.5°C) soils, but their viruses are also active and likely impacting soil microbial community structure and ecosystem biogeochemistry, as previously proposed [30, 31, 84, 85]. We observed an increase in vOTU richness and abundance from midyear to a full year of incubation, although vOTU evenness remained the same, likely due to a combination of proviruses replicating with active microbes and many generations of virions actively infecting newly active host(s). The few vOTUs that decreased in abundance or were present midyear and not detected after a full year could have been isotopically enriched and then some persisted in the soil as environmental DNA (eDNA), or as inert virions from internal nucleic acid errors (e.g., lethal mutations) or structural damage, or as competent virions that were no longer infective due to their host evolving resistance (e.g., the “host-virus arms race” that has been observed in other systems) [86].

While most viruses infect hosts within a species [87], having a broad host range can influence a virus’ ecological significance. Generalist viruses that can infect more than one species of the host are thought to have different abundance patterns, infection efficiency, movement within an ecosystem, and other unique attributes [88]. We identified fifteen generalists and four highly promiscuous generalist vOTUs that were predicted to infect hosts from different bacterial phyla. In addition, these four generalists were some of the most abundant vOTUs, suggesting that having a very broad host range may offer an advantage in these sub-freezing anoxic soils where the presence of active hosts may be more limited. Recently, Malki et al. [89] showed that four viruses from an oligotrophic lake in Michigan could infect bacteria across several phyla including Proteobacteria, Actinobacteriota, and Bacteroidota. Interestingly, the host phyla identified by Malki et al. [89] are the same bacterial phyla that our generalist vOTUs infected, with the addition of Firmicutes and Chloroflexota (also predicted by our network analyses, see Table S12; Fig. S4).

### Putative viral influence on winter biogeochemistry

Viruses shape the abundance, diversity, and metabolic outputs of their microbial hosts, and the impact viruses can have on an ecosystem depends heavily on the physiological state of the host, their defense mechanisms, and environmental conditions [27]. Arctic soils predominantly exist under anoxic sub-freezing temperature conditions, which affects microbial growth and metabolism as well as viral dynamics, especially in regard to reprogramming host metabolism [27, 90]. We detected many abundant viral genes that can help during an infection such as concanavalin A-like lectin/glucanases that may help in host recognition and attachment [91], methionine degradation which can help provide missing nutrients [92], and glycosyltransferases which can help viruses avoid host anti-viral mechanisms [83]. Viruses can also carry AMGs that are homologous to their host’s genes but may increase the host’s fitness. The metabolic functions and ubiquity of AMGs can vary by environment, with marine viruses commonly carrying AMGs for photosynthesis, central C metabolism, sulfur cycling, and nitrogen cycling [27]. Soil viruses, although understudied, appear to carry AMGs for polysaccharide degradation and sporulation [30, 31, 93], which can help their hosts breakdown complex carbohydrates into digestible sugars or protect their hosts when environmental conditions become unfavorable. In our cryoecosystem, we identified C degradation AMGs (e.g., galactose degradation and Xylan 1,4-beta-xylosidase) that could provide infected hosts with a fitness advantage for utilizing much-needed C sources, as well as genes for scavenging (e.g., ABC-2 type transport system) that may provide their host an avenue to acquire essential nutrients [94]. These genes show how viruses may remodel their host metabolic pathways during infection under simulated winter conditions, but additional AMG activity assays are needed to quantify their biogeochemical consequences.

To better understand the ecology and the roles viruses play in influencing the metabolic potential of the active microbial community in Arctic peat soils under winter conditions, we assessed the active bacterial phyla that were linked to active vOTUs. Firmicutes had the largest increase in abundance from midyear to a full year and had the most viral infections (both the number of linked active vOTUs and increase in vOTU abundance), consistent with the “kill-the-winner” theory described in aquatic systems (Figs. 4 and 5) [95]. In this Lotka–Volterra predator-prey framework, the most abundant host would become infected by viruses, leading to a decline in host abundance and an increase in the prevalence of their viruses, at least until microbial hosts with genetic resistance to the currently dominant viruses had time to respond. Another explanation for this parallel increase in abundance between Firmicutes and their linked vOTUs could be that the vOTUs were present as proviruses in their hosts’ genomes. This explanation is unlikely because lysogeny is thought to be prevalent at times of low host abundance [96] and the abundance of Firmicutes increased over the year incubation, and only about half of the vOTUs linked to Firmicutes were identified as temperate and even less were detected as proviruses. The dominance of Firmicutes makes sense, since this diverse bacterial phylum is known for adaptations to anoxic conditions, including fermentation and creating endospores [97]. In our samples, all active infected Firmicutes contained fermentation genes for the capacity to produce ethanol, lactate, or both (Table S9). Bacteroidota were also an active and frequently infected bacterial phylum that increased in abundance through time; of this group, all the infected MAGs shared taxonomic affiliation to the order Bacteroidales. Most of these bacteria are obligate anaerobes and known for their diverse arrays of carbohydrate-active enzymes arranged into polysaccharide utilization loci and fermentation [98, 99]. Many active MAGs within this group had the genomic capacity for polysaccharide degradation (e.g., ICE 1 encoded pectin degradation protein KdgF) and all encoded genes for lactate fermentation (Table S9). After 1 year under sub-freezing anoxic conditions, Bacteroidales had become the most abundant active lineage in our soils and were infected by the most abundant active vOTUs (#s 37, 41, 124, and 190; Table S3); none of which were predicted to be temperate viruses further supporting “kill-the-winner” theory and highlighting viruses influencing host winter activities. While the abundance of active Bacteroidota and Firmicutes populations were increasing, C mineralization to CO_2_ occurred steadily throughout the incubation and vOTUs linked to these populations carried several AMGs and other environmentally relevant genes that would support central C metabolism (Table S6). Together, these results suggest that these two bacterial lineages may play an important role in over-winter C loss from these Artic peat soils, and the viruses that infect them likely shape both their population dynamics and functional impact.

Another way viruses may impact soil biogeochemistry is by infecting hosts that occupy different metabolic niches. The Patescibacteria we identified (part of the Candidate phyla radiation; CPR) are known for their ultrasmall cell size with reduced genomes and most likely have a symbiotic relationship with Acidobacteriota [100, 101] and possibly Bacteroidota [102], suggesting direct interactions between these lineages in our cryoecosystem. Their relationships with Acidobacteriota appear to be broad and induce different physiological changes as seen with their varying growth and crash phases which affect host metabolic outputs [100]. Patescibacteria are thought to resist phage infection by lacking or reducing the number of potential phage receptors on their cell membrane [103], but in our study, it was notably one of the most infected lineages and had none of the previously reported phage receptors (Table S9). The prevalence of these infections may be the result of their interactions with Bacteroidota. One member of the Patescibacteria was linked to an unlabeled vOTU with a CRISPR spacer match, suggesting this adapted immune system element was successful at protecting the host from infection. Typical CRISPRs are rarely found in CPR bacteria, and recent work suggests this may be due to this group using a compact CRISPR-CasY system that is highly divergent to typical CRISPR systems [104]. Another infected and active bacterial phylum we observed was Proteobacteria, with only one active MAG from the class Micavibrionales. Little is known about this clade, and even less about their viruses, because they have an obligate epibiotic lifestyle where they feed on other organisms to survive, making them difficult to culture. A recent survey of 14 soils across North America (including one sample from Arctic Alaska) revealed obligate predatory bacteria grew faster and assimilated about two-fold more C than nonpredatory bacteria [105]. These predatory bacteria may be active under winter conditions by either consuming non-active or dead cells, or they may benefit from attaching to a host that can utilize alternative energy sources and recalcitrant organic matter [106]. Schimel and Mikan [107] showed that at freezing and sub-freezing temperatures most of the total soil respiration came from recycling of microbial biomass and products, and not from detritus or soil organic matter, suggesting these predatory bacteria and their viruses may have a larger role in dictating the microbial community structure and C flow in Arctic soils.

### Challenges associated with identifying virus “activity”

Heavy-water SIP has proven to be a robust method for identifying metabolically active microbes in soils [37, 38, 41]. In many ways, this approach produces more direct evidence of growth activity compared to other techniques that track activity such as RNA-to-DNA ratios, rRNA levels, bioorthogonal non-nanonical amino acid tagging (BONCAT), or other stable isotopes (e.g., ^13^C) because active microbes synthesize DNA when their cells divide, incorporating ^18^O, and therefore, the DNA of all actively replicating microbes is labeled. Data from RNA studies can be hard to interpret as RNA levels often do not correlate with growth and have weak or no correlation with protein levels [108–110]. Compared to other isotopes as tracers, ^18^O labeling via heavy-water SIP does not rely on the microbe’s C use efficiency or prior knowledge of the microbe’s substrate preference [42].

The application of isotope tracers for direct assessment of activity is not as straightforward for viruses as it is for their microbial hosts, and worthy of reasoned consideration. Characterizing activity for viruses is quite different compared to “free-living” organisms because of their different infection cycles, their lack of metabolism, and the many states in which they can be present [18]. One of the main reasons to identify active entities is to help quantify their impact on their hosts and their environment, with the assumption that inactive entities have less of an impact but are still important [111]. For a microbe, this may be true, but for a virus, there is a range in magnitude of impact for different host metabolic states that depends heavily on the infection cycle. Assessing the prevalence of an infection cycle, however, is challenging due to difficulties in quantifying lysogeny or virion abundance, burst size, diversity, and ecology [24].

Arctic soils that predominantly exist under anoxic sub-freezing temperature conditions might be generally considered a harsh environment, which would limit microbial growth. For this reason, we hypothesized most of our viruses would be temperate [24, 112] and they would be detected as proviruses. We did see a high rate of lysogeny (43%), and in support of our hypotheses, the majority (80%) of temperate viruses were present as proviruses. The temperate viruses linked to microbial hosts spanning seven bacterial phyla, but almost half were linked to unlabeled MAGs from the Actinobacteriota phylum. Further, half of the active vOTUs linked to active MAGs were temperate viruses. We hypothesize the increased incidence of temperate viruses is linked to low host abundances and environmental conditions, therefore increasing the potential for sporadic viral infection.

Temperate viruses can undergo lytic infection, where activity is identified by progeny viruses, or lysogenic infection, where activity is difficult to assess. A temperate virus that is undergoing lysogenic infection (present as a provirus) during a heavy-water SIP incubation would become isotopically enriched and depend on its host’s division rate for abundance. Active viruses undergoing lysogenic infection need to be distinguished from viruses undergoing lytic infection because the effect of proviruses on the host metabolism (and therefore ecosystem) will not be as pronounced. This is because proviruses do not shut down and redirect host metabolism for progeny production during the lysogenic cycle as compared to the lytic cycle. Proviruses may also be labeled, but not currently active if they are proviral remnants of a past infection [113]. These remnants have no negative impact on host metabolism (beyond occupying genome space) but may confer some advantage as a gene transfer agent [24] or by contributing virulence factors which can still impact host physiology and metabolism [90]. SIP-enabled metagenomics alone cannot unequivocally identify a virus’ state (e.g., in maintenance mode or a remnant), making it currently difficult to fully assess viral activity. Combining SIP-metagenomics with other approaches—such as a SIP-virome or induction assays—may identify vOTUs that have undergone lytic infection and therefore provide a more holistic view of vOTU dynamics.

## Conclusions

Winter C losses in Arctic peat soils are estimated to be significant and growing, but the mechanisms that drive these losses are poorly understood. We identified active bacteria and dsDNA viruses in conditions simulating the long winter months in northern peatland soils (anoxic and −1.5°C), and these active populations drive significant CO_2_ fluxes. Our approach using heavy-water SIP-targeted metagenomics allowed us to move beyond a general catalog of the genetic repertoire of these soil communities and expose the specific population-level dynamics and functional capacities of the active bacterial and dsDNA viral community. Given the high number and abundance of unlabeled bacteria, viruses, and virus-host linkages, in a more traditional analysis (without SIP), these quiescent populations would likely have masked the characterization of these soils’ active bacteria and viruses. Despite the lack of oxygen and sub-freezing temperatures in these peat soils, many bacteria and viruses (both temperate and virulent) are active and appear to be engaged in a high level of biotic interactions and biogeochemical processing.

## Supplementary Information

**Additional file 1: Supplementary text**. Placing vOTUs in the context of global viruses. Generation of gene-sharing networks.

**Additional file 2: Supplementary Fig. S1**. An overview of methods. **Supplementary Fig. S2**. A gene-sharing network with RefSeq viruses. **Supplementary Fig. S3**. vOTUs observed in the SIP fractions. **Supplementary Fig. S4**. A gene-sharing network with RefSeq and Genbank viruses.

**Additional file 3: Supplementary Table S1**. Sample information. **Supplementary Table 2**: Contigs consumed in vOTUs. **Supplementary Table 3**: List of all vOTUs derived from this study and their statistics. **Supplementary Table S4**. vOTU taxonomy with RefSeq (v85). **Supplementary Table S5**. Multiphate annotations. **Supplementary Table S6**. Putative AMGs and viral genes of interest. **Supplementary Table S7**. Temperate viruses. **Supplementary Table S8**. MAG information. **Supplementary Table S9**. MAG genes. **Supplementary Table S10**. vOTU-MAG CRISPR spacer matches. **Supplementary Table S11**. vOTU-MAG BLASTn matches. **Supplementary Table 12**. vOTU taxonomy with RefSeq (v85) and Genbank.

## Data Availability

The 23 metagenomes generated in this study were deposited to NCBI under BioProject identifier (ID) PRJNA634918 with BioSample information included in Table S1. Figures were generated with Microsoft Excel and R, using packages Vegan for diversity, pheatmap for heat maps, ggplot for virus:host abundance chart, and ggraph for virus-host network. *T* tests and linear regressions were performed using the data analysis package in Excel.

## Abbreviations

APEX: Alaska Permafrost Experiment
AMG: Auxiliary metabolic gene
BLAST: Basic Local Alignment Search Tool
C: Carbon
CO_2_: Carbon dioxide
CRISPR: Clustered Regularly Interspaced Short Palindromic Repeat
DNA: Deoxyribonucleic acid
GTDB: Genome Taxonomy Database
H_2_^18^O: Heavy water
KEGG: Kyoto Encyclopedia of Genes and Genomes
MAG: Metagenome-assembled genome
RNA: Ribonucleic acid
SIP: Stable isotope probing
TE: Tris-ethylenediaminetetraacetic acid
vOTU: Viral Operational Taxonomic Unit
