# Supplementary figures and images for: Active virus-host interactions at sub-freezing temperatures in Arctic peat soil
Source: Microbiome. 2021 Oct 18;9:208. doi: 10.1186/s40168-021-01154-2 (PMC8522061; doi:10.1186/s40168-021-01154-2)

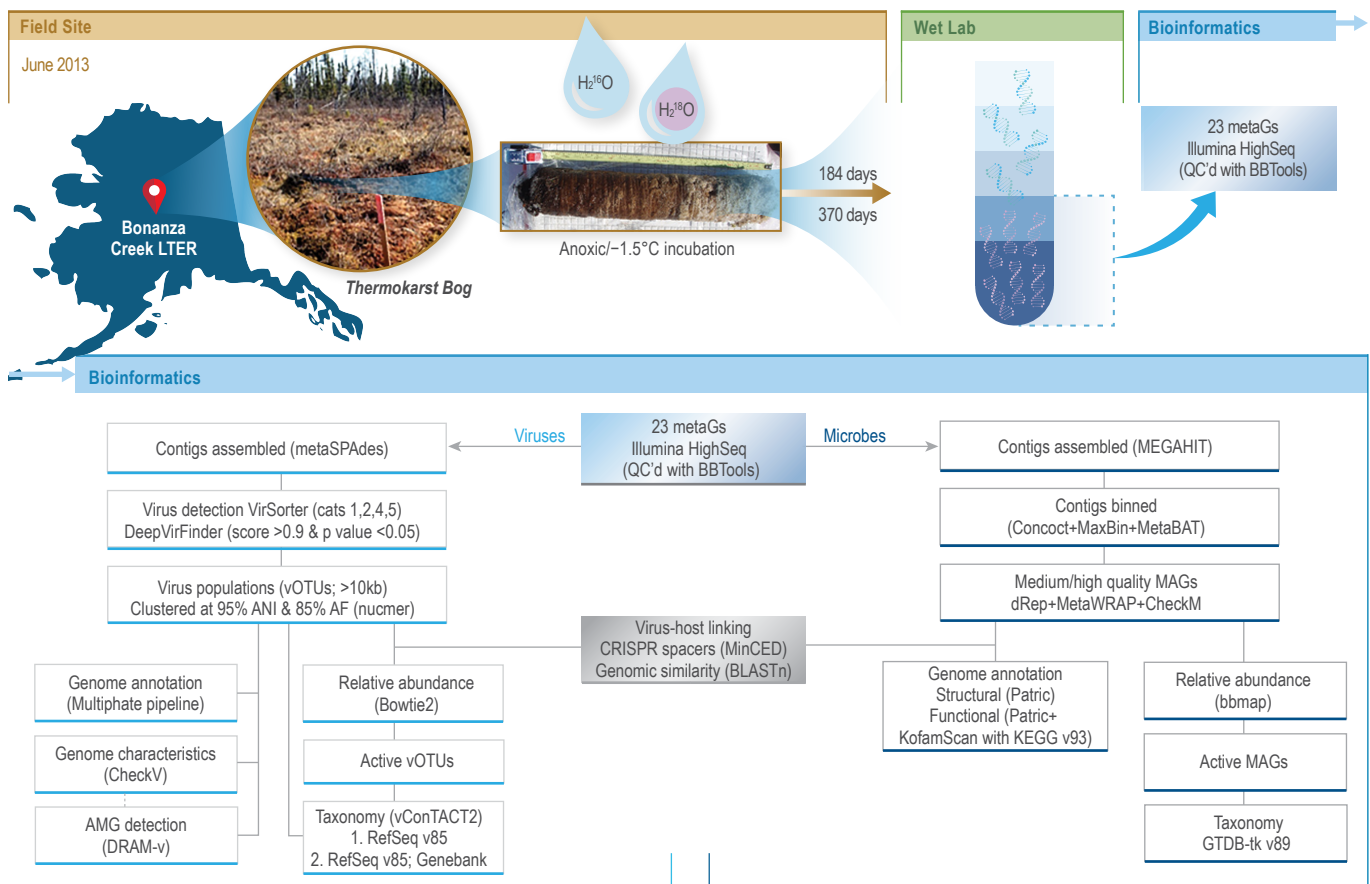

Supplement: Supplementary file 3 — Additional file 2: Supplementary Fig. S1. An overview of methods. Supplementary Fig. S2. A gene-sharing network with RefSeq viruses. Supplementary Fig. S3. vOTUs observed in the SIP fractions. Supplementary Fig. S4. A gene-sharing network with RefSeq and Genbank viruses. [file 40168_2021_1154_MOESM3_ESM.zip › FigureS1.pdf]

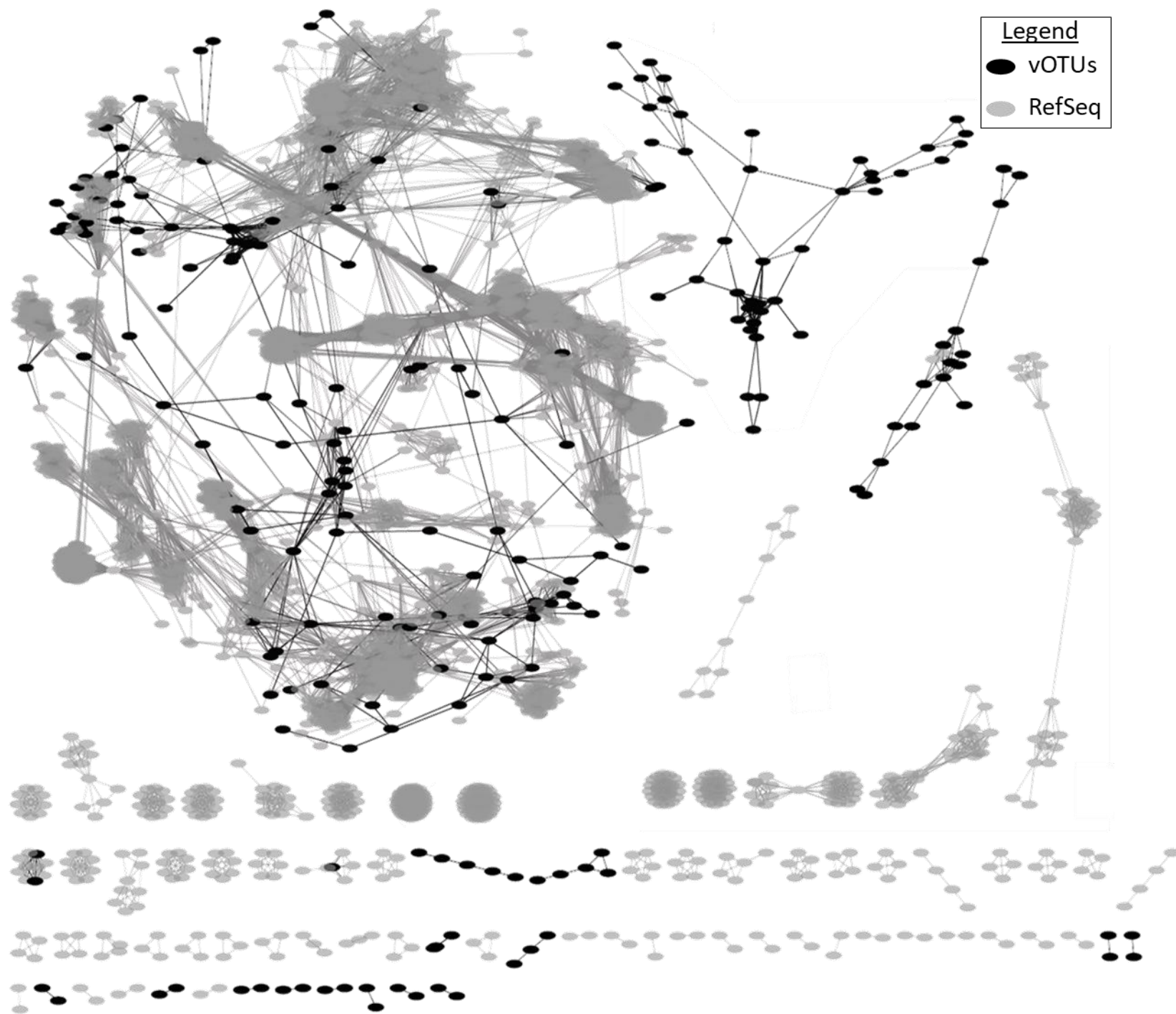

Supplement: Supplementary file 3 — Additional file 2: Supplementary Fig. S1. An overview of methods. Supplementary Fig. S2. A gene-sharing network with RefSeq viruses. Supplementary Fig. S3. vOTUs observed in the SIP fractions. Supplementary Fig. S4. A gene-sharing network with RefSeq and Genbank viruses. [file 40168_2021_1154_MOESM3_ESM.zip › FigureS2.pdf]

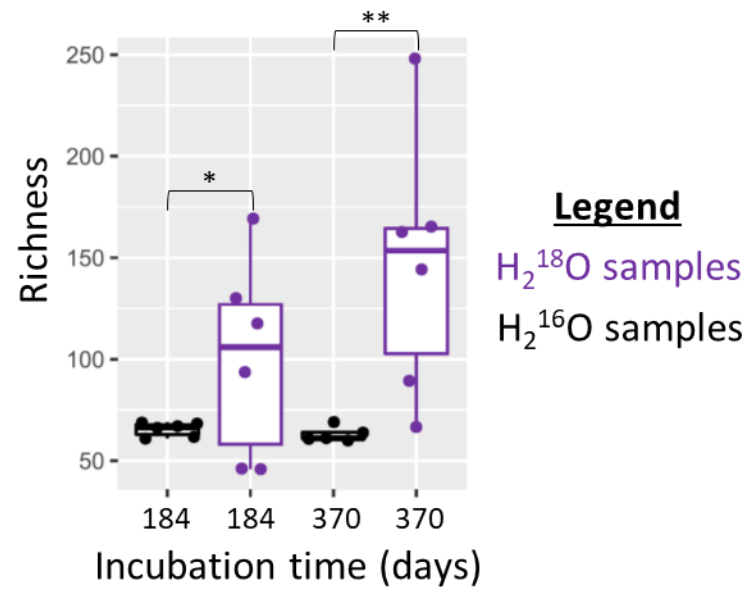

Supplement: Supplementary file 3 — Additional file 2: Supplementary Fig. S1. An overview of methods. Supplementary Fig. S2. A gene-sharing network with RefSeq viruses. Supplementary Fig. S3. vOTUs observed in the SIP fractions. Supplementary Fig. S4. A gene-sharing network with RefSeq and Genbank viruses. [file 40168_2021_1154_MOESM3_ESM.zip › FigureS3.pdf]

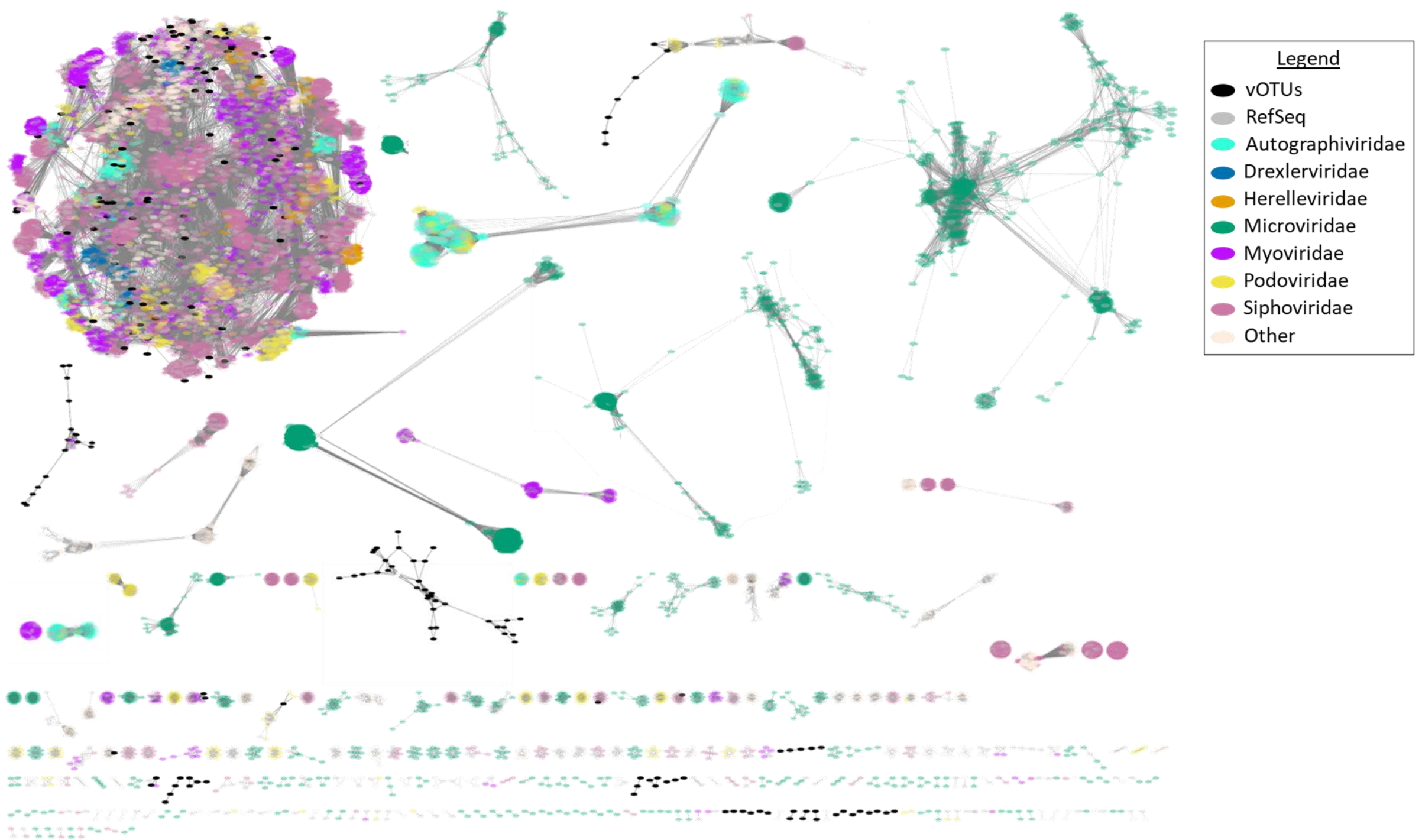

Supplement: Supplementary file 3 — Additional file 2: Supplementary Fig. S1. An overview of methods. Supplementary Fig. S2. A gene-sharing network with RefSeq viruses. Supplementary Fig. S3. vOTUs observed in the SIP fractions. Supplementary Fig. S4. A gene-sharing network with RefSeq and Genbank viruses. [file 40168_2021_1154_MOESM3_ESM.zip › FigureS4.pdf]
